# Characteristics of air–liquid heat and mass transfer in a bubble column humidifier

**DOI:** 10.1016/j.applthermaleng.2022.118240

**Published:** 2022-06-05

**Authors:** Elias Eder, Sandro Hiller, Dieter Brüggemann, Markus Preißinger

**Affiliations:** a illwerke vkw Endowed Professorship for Energy Efficiency, Research Center Energy, Vorarlberg University of Applied Sciences, 6850 Dornbirn, Austria; b Chair of Engineering Thermodynamics and Transport Processes (LTTT), Center of Energy Technology (ZET), University of Bayreuth, 95440 Bayreuth, Germany

**Keywords:** Bubble column humidifier, Humidification–dehumidification, Desalination, Air–liquid heat transfer, Air–liquid mass transfer

## Abstract

Bubble columns are recently used for the humidification of air in water treatment systems and fuel cells. They are well applicable due to their excellent heat and mass transfer and their low technical complexity. To design and operate such devices with high efficiency, the humidification process and the impact of the operating parameters need to be understood to a sufficient degree. To extend this knowledge, we use a refined and novel method to determine the volumetric air–liquid heat and mass transfer coefficients and the humidifier efficiency for various parametric settings. The volumetric transfer coefficients increase with both of the superficial air velocity and the liquid temperature. It is further shown that the decrease of vapor pressure with an increase of the salinity results in a corresponding decrease in the outlet humidity ratio. In contrast to previous studies, liquid heights smaller than 0.1 m are investigated and significant changes in the humidifier efficiency are seen in this range. We present the expected humidifier efficiency with respect to the superficial air velocity and the liquid height in an efficiency chart, such that optimal operating conditions can be determined. Based on this efficiency chart, recommendations for industrial applications as well as future scientific challenges are drawn.

## Graphical abstract

**Figure:**
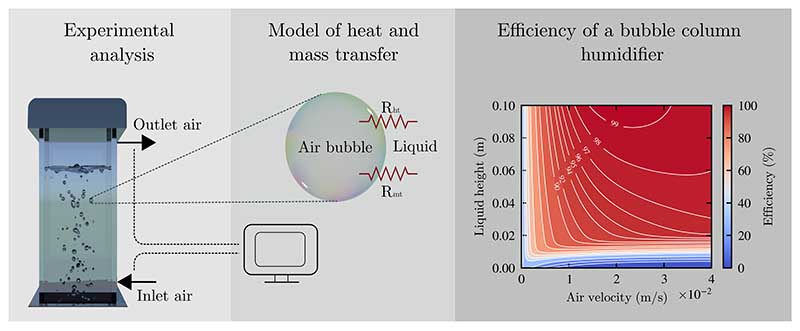

## 1 Introduction

Today’s increasing water scarcity and the growing trend towards renewable energy sources have encouraged the development of a new application for bubble column reactors: *the humidification of air*. Bubble columns have unique advantages for humidification. They have high heat and mass transfer rates, are technically simple and do not require packing materials. Thus, they are superior to conventional humidifiers [1–4]. Nonetheless, the design and operation of these devices are not yet optimized. To better understand the humidification process, a comprehensive model of the air–liquid heat and mass transfer is needed.

Traditionally, bubble column reactors are used for gas–liquid reactions in chemical process engineering. Main applications are absorption processes [5–7], Fischer–Tropsch synthesis of synthetic fuels [8–10], or biochemical reactions [11,12]. The majority of these processes involve endothermic or exothermic reactions that need supply or removal of heat. Therefore, the heat transfer between submerged objects in the column and the liquid–gas mixture has been studied to a vast extent [13–15]. Mass transfer was studied frequently for typical absorption processes [16,17].

More recently, bubble column humidifiers (BCHs) are applied (1) in the desalination of seawater or brackish water by the humidification–dehumidification process (HDH) [18–20] and (2) in membrane hydration for fuel cells [21–23]. As the degree of evaporation into the air stream is of immanent relevance for the efficiency of these applications, the direct contact air–liquid heat and mass transfer need to be investigated.

Several in-depth studies have been carried out on the impact of the main operating parameters in BCHs [24–28]. However, these studies also have several gaps and drawbacks: (1) Heat and mass transfer characteristics between the air stream and the continuous liquid phase are rarely analyzed, (2) the air state at the humidifier outlet is often not investigated systematically and (3) different conclusions are drawn regarding the impact of operating parameters as different operating ranges are investigated. In particular, the impact of liquid height is often characterized insufficiently. However, minimizing the liquid height allows for a more efficient operation of BCHs and is therefore of great interest.

In a previous study, we have already tried to overcome some of these shortcomings by investigating the operating parameters in a BCH [29]. We found that productivity changes in the system can be traced back to changes in the air temperature at the humidifier outlet. However, we have not systematically investigated the impact of all the main operating parameters in relevant ranges, nor have we presented a model that describes the governing mechanisms of humidification in a bubble column.

A first attempt in modeling humidification and dehumidification in bubble columns has been presented by Narayan et al. [30]. They developed an analytical model for heat and mass transfer in a bubble column dehumidifier that models the mass transfer coefficient using film theory for the gas-side resistance and penetration theory for the liquid-side resistance. Subsequently, they calculated the heat transfer coefficient for the heat sink using the Lewis factor analogy for combined heat and mass transfer devices. Heat flux transferred to the cooling coil was generally predicted well with underestimations of less than 20% of the experimental values.

Zizka et al. [31] investigated gas–liquid heat transfer in a bubble column with dependence on the aerator design and the superficial air velocity by applying an energy balance to the liquid. They presented correlations for the volumetric heat transfer coefficient and were able to show that the impact of the liquid height on the volumetric heat transfer coefficient can be compensated by using aeration as a measure of the airflow. However, they only investigated liquid heights higher than 0.5 m.

Katz et al. [32] modeled the heat transfer rates between the air stream and the liquid phase in a transient evaporative cooling process and assumed the air to be at the liquid temperature and saturated after humidification. Air at the liquid surface was assumed to be at the liquid temperature based on their measurement of 40 cm above the liquid surface. They also reported an issue with the formation of liquid droplets on their humidity sensor which did not allow an accurate measurement of the air state at the humidifier outlet.

Inaba et al. [33] applied an energy balance to the air stream to estimate the heat and mass transfer between air and liquid. They correlated nondimensional expressions for the heat and mass transfer coefficients. In their study, the impact of the liquid height on the heat and mass transfer coefficients was not investigated.

A model of the air–liquid heat and mass transfer, including the dependence on all main operating parameters in relevant ranges, is still missing. Such a model is developed in our study. To evaluate it, exact knowledge of the air state at the outlet of a BCH is crucial. An optimized test setup and an optimized modeling method are used for this purpose. The unique novelties of our study are:

- The liquid temperature, the superficial air velocity and the liquid height can be adjusted and maintained in their relevant ranges. In particular, the study of low liquid heights is of great interest in this matter.
- The temperature of the outlet air stream is measured directly at the liquid surface and at the humidifier outlet to determine the outlet air state at different positions.
- The temperature and humidity of the outlet air are also measured using a humidity sensor. The formation of water droplets on the humidity sensor is avoided by using a heating line before the sensor.
- The volumetric transfer coefficients are determined by nonlinear regression of multiple measurements instead of the calculation based on single values. By using nonlinear regression, a false dependence of the volumetric transfer coefficients on the liquid height can be avoided.

The operating parameters investigated in this study are the liquid temperature, the superficial air velocity and the liquid height. As desalination is the most crucial application of BCHs, we also investigate the salinity of the liquid as an operating parameter. Correlations are derived for the volumetric heat and mass transfer coefficients which drive the humidification process and recommendations are given for the efficient design and operation of BCHs. Therefore, the presented work extends the understanding of heat and mass transfer in a bubble column by defining relevant parameters and their respective ranges for efficient humidification of air.

## 2 Analytical model

An analytical model and experimental analysis of the humidification are combined for various parametric settings to determine the volumetric heat and mass transfer coefficients.

When in contact with water, air is humidified by evaporation due to concentration gradients between the gas–liquid contact area and the air bubble’s moist air mixture. With respect to the evaporation of water, latent heat is transferred. Sensible heat is also transferred as it is induced by the temperature difference between the air stream and the liquid phase.

**Fig. 1:**
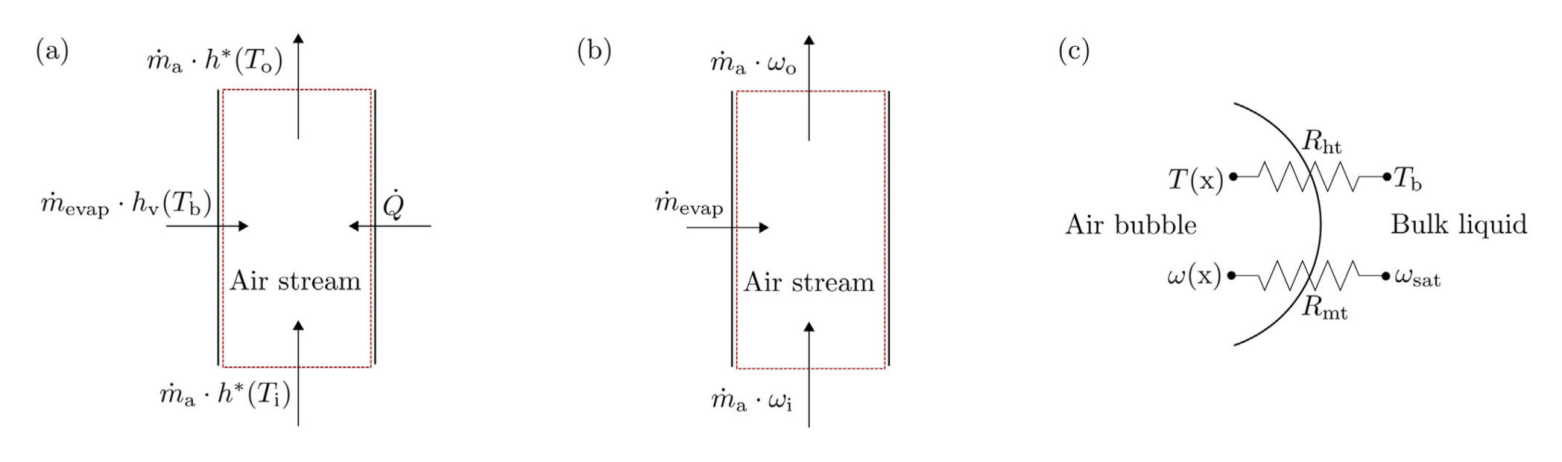
(a) Global energy balance of the air stream, (b) global mass balance of the water vapor in the air stream and (c) heat and mass transfer resistances between the air–vapor mixture and the bulk liquid for a single bubble.

The air–liquid heat and mass transfer in the BCH is modeled based on a control volume of the air stream (see Fig. 1). The following assumptions are made for the developed model:

- The liquid phase is ideally mixed and therefore isothermal.
- Spatial variations in temperature or salinity are neglected.
- Changes in the specific heat capacities of air and water vapor are negligible within the investigated parametric range.

### 2.1 Global system balances

In Fig. 1(a) a control volume with an energy balance of the air stream, (b) a control volume with a mass balance of the water vapor in the air stream and (c) an air bubble with the associated heat and mass transfer resistances *R*_ht_ and *R*_mt_ are shown.

The overall heat flow $\dot{Q}$ transferred to the air stream can be expressed by Eq. (1) with ${\dot{Q}}_{\text{sens}}$ denoting the sensible and ${\dot{Q}}_{\text{lat}}$ denoting the latent heat flow:

(1) $$\dot{Q}={\dot{Q}}_{\text{sens}}+{\dot{Q}}_{\text{lat}}$$

In accordance with Fig. 1(a), the heat flow can also be expressed by Eq. (2):

(2) $$\dot{Q}={\dot{m}}_{\text{a}}\cdot \left[h^{*}\left(T_{\text{o}}\right)-h^{*}\left(T_{i}\right)\right]-{\dot{m}}_{\text{evap}}\cdot h_{\text{v}}\left(T_{\text{b}}\right),$$

with ${\dot{m}}_{\text{a}}$ representing the dry air mass flow, ${\dot{m}}_{\text{evap}}$ representing the amount of water evaporated, h* and *h*_v_ representing the specific enthalpy of moist air and water vapor, *T*_o_ and *T*_i_ representing the air temperatures at the liquid surface and at the bubble column inlet, and *T*_b_ representing the bulk liquid temperature.

The specific enthalpy of moist air h* at a specific temperature *T* is defined as follows:

(3) $$h^{*}\left(T\right)=c_{\text{p},\text{a}}\cdot T+\omega \cdot \left[\Delta h_{\text{v}}+c_{\text{p},\text{v}}\cdot T\right],$$

with *c*_p,a_ and *c*_p,v_ representing the specific heat capacities of air and water vapor, *ω* representing the humidity ratio of moist air, Δ*h*_v_ representing the enthalpy of vaporization, and *T* representing the air temperature.

Combining Eqs. (2) and (3) gives an expression for both the latent and sensible heat flow to the air stream (Eqs. (4) and (5)):

(4) $${\dot{Q}}_{\text{sens}}={\dot{m}}_{\text{a}}\cdot \left[c_{\text{p},\text{a}}\cdot \left(T_{\text{o}}-T_{\text{i}}\right)+\omega_{\text{i}}\cdot c_{\text{p},\text{v}}\cdot \left(T_{\text{o}}-T_{\text{i}}\right)+\left(\omega_{\text{o}}-\omega_{\text{i}}\right)\cdot c_{\text{p},\text{v}}\cdot \left(T_{\text{o}}-T_{\text{b}}\right)\right]$$

and

(5) $${\dot{Q}}_{\text{lat}}={\dot{m}}_{\text{evap}}\cdot \Delta h_{\text{v}},$$

with *ω*_o_ and *ω*_i_ denoting the humidity ratios of air at the liquid surface and humidifier inlet, respectively.

It can be seen that ${\dot{Q}}_{\text{sens}}$ consists of three individual heat flows representing (1) the temperature change of dry inlet air, (2) the temperature change of inlet water vapor and (3) the temperature change of evaporated water.

The sensible heat transferred to the air stream ${\dot{Q}}_{\text{sens}}$ can also be expressed by Eq. (6):

(6) $${\dot{Q}}_{\text{sens}}=h_{\text{t}}\cdot a_{\text{s}}\cdot V\cdot \Delta T,$$

with *h*_t_ representing the heat transfer coefficient, *a*_s_ representing the specific interfacial area, *V* representing the volume of the aerated bubble column, and *ΔT* representing the temperature difference which drives the sensible heat transfer.

By applying a global mass balance to the air stream in the humidifier (see Fig. 1(b)), the amount of water evaporated can be expressed using Eq. (7):

(7) $${\dot{m}}_{\text{evap}}={\dot{m}}_{\text{a}}\cdot \left(\omega_{\text{o}}-\omega_{\text{i}}\right)$$

The amount of water evaporated can also be expressed as

(8) $${\dot{m}}_{\text{evap}}=k_{\text{t}}\cdot a_{\text{s}}\cdot V\cdot \Delta \omega ,$$

with *k*_t_ representing the mass transfer coefficient, *a*_s_ representing the specific interfacial area, *V* representing the volume of the aerated bubble column, and *Δω* representing the characteristic humidity ratio difference which drives the evaporation and therefore the latent heat transfer.

With knowledge of the characteristic temperature difference *ΔT* and humidity ratio difference *Δω*, the volumetric heat transfer coefficient *h*_t_*a*_s_ can be determined using Eqs. (4) and (6) and the volumetric mass transfer coefficient *k*_t_*a*_s_ can be determined using Eqs. (7) and (8).

Several researchers [33,34] used Eqs. (4) to (8) for calculating the volumetric heat and mass transfer coefficients based on single measurement values. In this study we show that this approach is not suitable for accurate characterizations of the heat and mass transfer and present an improved methodology. We use the analytically determined humidity ratio profile and temperature profile of the air stream in the bubble column for this novel method. These are derived based on local mass and energy balances in the following sections.

### 2.2 Humidity ratio profile

**Fig. 2:**
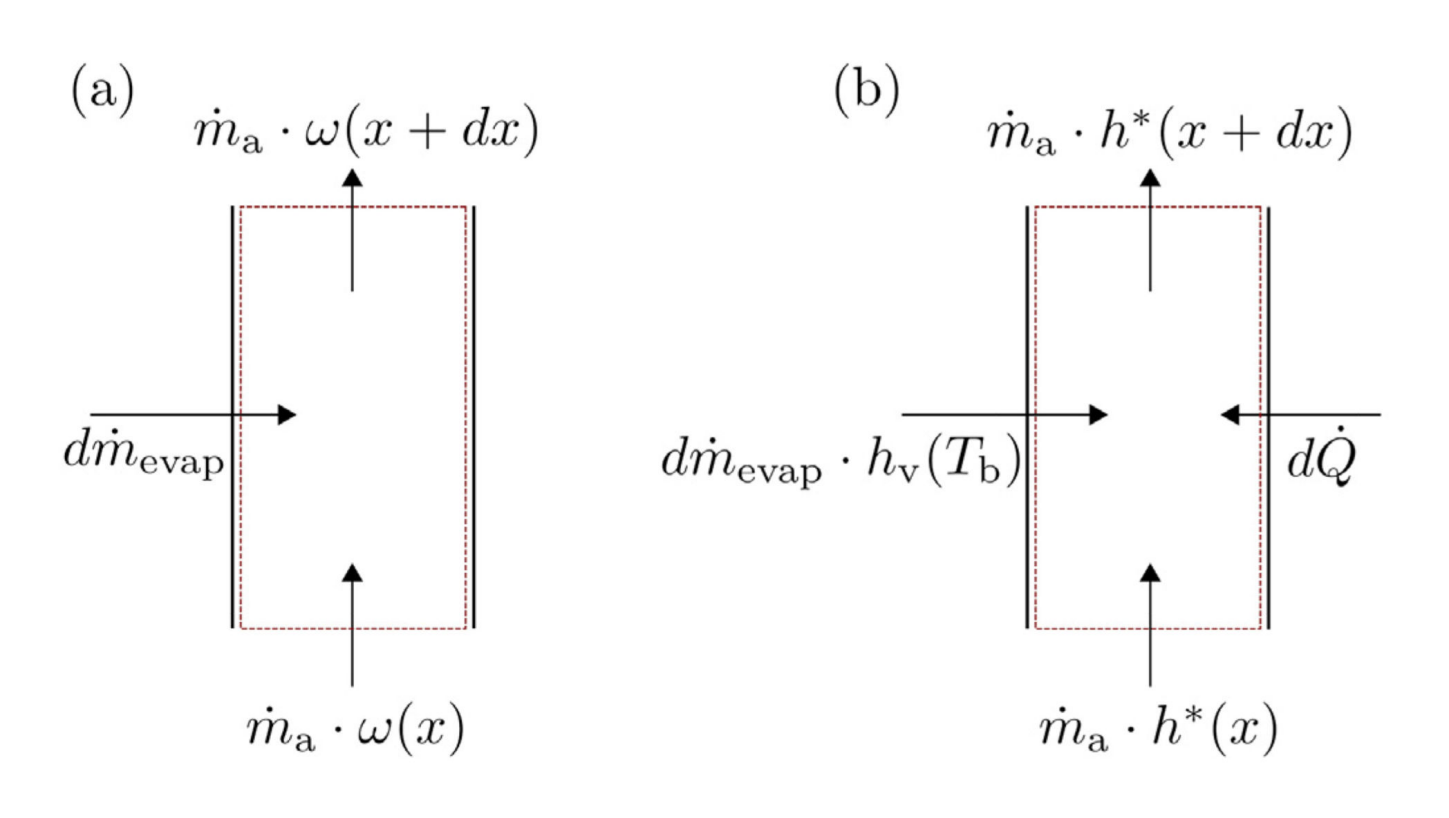
(a) Infinitesimal mass balance of water vapor contained in the air stream and (b) infinitesimal energy balance of the air stream in the bubble column.

To derive the humidity ratio of air as a function of the vertical position *x* in the bubble column, a mass balance is applied to an infinitesimal control volume. A mass balance and an energy balance are depicted for a control volume with length *dx* in Fig. 2(a) and (b), respectively.

The mass balance from Fig. 2(a) yields Eq. (9):

(9) $$d{\dot{m}}_{\text{evap}}={\dot{m}}_{\text{a}}\cdot \left[\frac{d\omega }{dx}\cdot dx\right]$$

The differential amount of water evaporating can also be expressed by Eq. (10):

(10) $$d{\dot{m}}_{\text{evap}}=k_{\text{t}}\cdot P\cdot dx\cdot \left[\omega_{\text{sat}}-\omega \left(x\right)\right],$$

with *k*_t_ referring to the mass transfer coefficient, *P* referring to the equivalent perimeter of the control volume, and *ω*_sat_ referring to the maximum possible humidity ratio of the air stream (which is equal to the air stream reaching liquid temperature and being saturated).

Combining Eqs. (9) and (10), we obtain

(11) $$\frac{d\omega \left(x\right)}{dx}+\frac{k_{\text{t}}\cdot P}{{\dot{m}}_{\text{a}}}\cdot \omega \left(x\right)=\frac{k_{\text{t}}\cdot P}{{\dot{m}}_{\text{a}}}\cdot \omega_{\text{sat}}.$$

Eq. (11) can be solved analytically and leads to

(12) $$\omega \left(x\right)=\omega_{\text{sat}}+\left(\omega_{i}-\omega_{\text{sat}}\right)\cdot e^{-a\cdot x},$$

with parameter *a*

(13) $$a=\frac{k_{\text{t}}\cdot a_{\text{s}}\cdot A_{\text{c}}}{{\dot{m}}_{\text{a}}},$$

where the equivalent perimeter of the control volume *P* is replaced by the product of the specific interfacial area and the cross-sectional area of the bubble column (*P* = *a*_s_ ⋅ *A*_c_).

It can be shown that Eq. (12) is in its mathematical form equivalent to a nondimensional temperature profile for a heat exchanger in contact with an isothermal bulk liquid. It is therefore evident that the concentration difference driving mass transfer is a mean logarithmic humidity ratio difference. The mean logarithmic humidity ratio difference is in agreement with the heat and mass transfer models of other researchers [33,35].

With knowledge of parameter *a* (Eq. (13)), it is possible to determine the volumetric mass transfer coefficient *k*_t_*a*_s_.

### 2.3 Temperature profile

According to Fig. 2(b), applying the energy balance to the air stream leads to Eq. (14):

(14) $$d\dot{Q}={\dot{m}}_{\text{a}}\cdot \left[h^{*}\left(x+dx\right)-h^{*}\left(x\right)\right]-d{\dot{m}}_{\text{evap}}\cdot h_{\text{v}}\left(T_{\text{b}}\right)$$

Using Eq. (14), the analytical temperature profile of the air stream in the bubble column can be deduced. Details can be found in a publication of Tow and Lienhard [35], who previously derived this temperature profile.

The analytical temperature profile is given in Eq. (15). In contrast to the standard form of the mean logarithmic temperature difference, changes in the heat capacity rates are considered here:

(15) $$T\left(x\right)=T_{\text{b}}+\left(T_{\text{i}}-T_{\text{b}}\right)\cdot e^{-b\cdot x}\cdot {\left[\frac{C_{\text{i}}^{*}}{C^{*}\left(x\right)}\right]}^{\frac{b}{a}+1}$$

with

(16) $$b=\frac{h_{\text{t}}\cdot a_{\text{s}}\cdot A_{\text{c}}}{C_{\text{sat}}^{*}}$$

and

(17) $$C_{\text{i}}^{*}={\dot{m}}_{\text{a}}\cdot \left(c_{\text{p},\text{a}}+\omega_{\text{i}}\cdot c_{\text{p},\text{v}}\right)$$

(18) $$C_{\text{sat}}^{*}={\dot{m}}_{\text{a}}\cdot \left(c_{\text{p},\text{a}}+\omega_{\text{sat}}\cdot c_{\text{p},\text{v}}\right)$$

(19) $$C^{*}\left(x\right)=C_{\text{sat}}^{*}+\left[C_{\text{i}}^{*}-C_{\text{sat}}^{*}\right]\cdot e^{-a\cdot x}$$

In Eqs. (15) to (19), C* is referring to the heat capacity rate of the moist air stream. With knowledge of parameter *b*, it is possible to determine the volumetric heat transfer coefficient *h*_t_*a*_s_.

### 2.4 Improved methodology

**Fig. 3:**
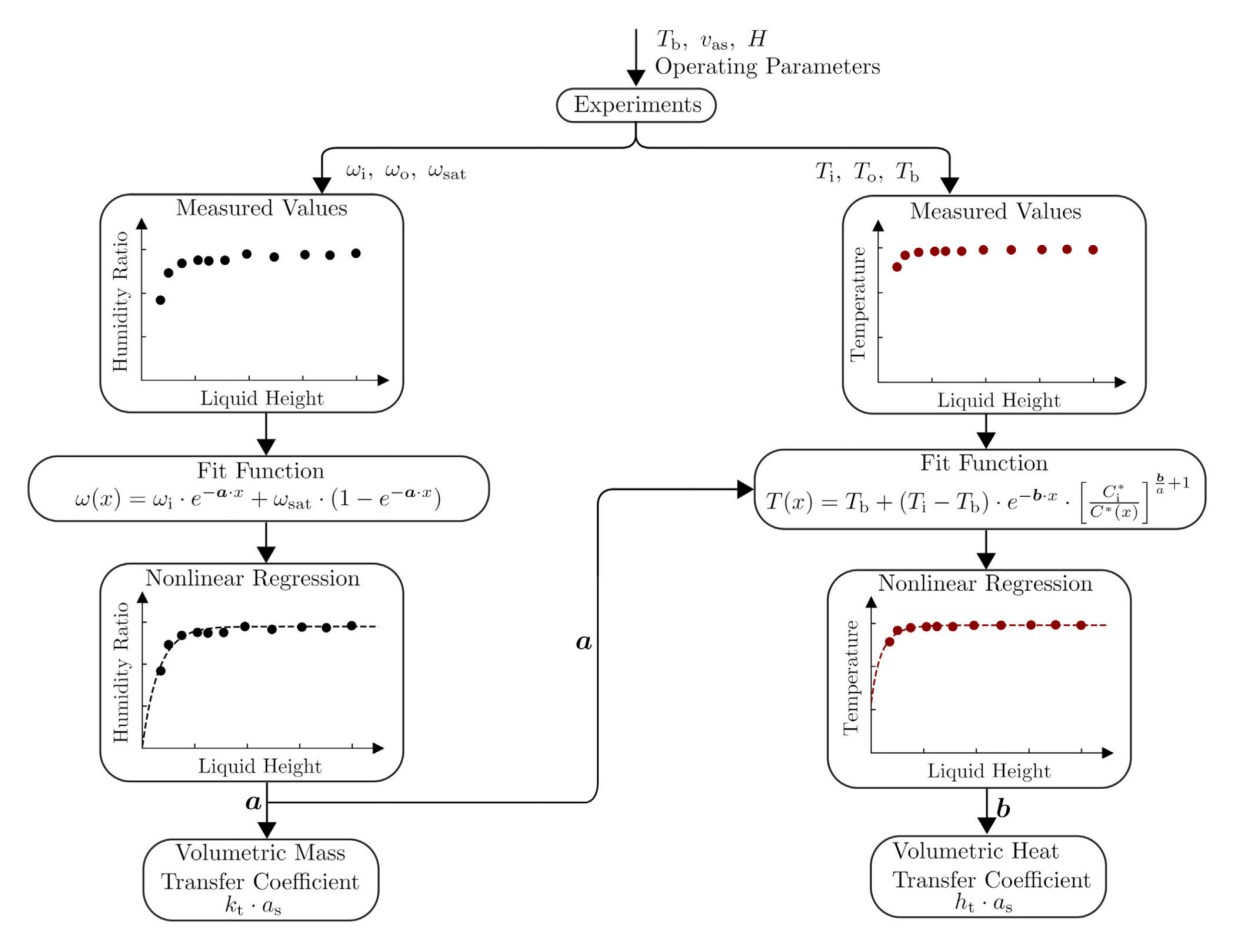
Combination of experiments and nonlinear regression to determine the volumetric transfer coefficients.

In Fig. 3, a flow chart is given that describes our methodology to determine the volumetric transfer coefficients.

The method of determining the volumetric transfer coefficients in accordance with Fig. 3 is defined as follows:

- The bulk liquid temperature *T*_b_, the superficial air velocity *υ*_as_ and the liquid height *H* are varied within the experiments to generate data regarding the air state (humidity ratio and temperature) at the humidifier inlet and outlet.
- Using these data and the fit function (Eq. (12)), nonlinear regression is used to determine the relevant parameter *a* and to subsequently calculate the volumetric mass transfer coefficient *k*_t_*a*_s_.
- To determine parameter *b* and therefore the volumetric heat transfer coefficient *h*_t_*a*_s_, the same procedure is used with the fit function Eq. (15). Additionally, parameter *a* is needed, as it is contained in Eq. (15).

### 2.5 Lewis factor and humidifier efficiency

The relative rates of heat and mass transfer in simultaneous heat and mass exchangers and generally in evaporative processes are usually evaluated using the Lewis factor *Le*_f_ [36]. The minimum Lewis factor in a BCH can be calculated by

(20) $$Le_{\text{f}}=\frac{h_{\text{t}}}{k_{\text{t}}\cdot c_{\text{p},\text{sat}}^{*}},$$

with

(21) $$c_{\text{p},\text{sat}}^{*}=c_{\text{p},\text{a}}+\omega_{\text{sat}}\cdot c_{\text{p},\text{v}}.$$

Typical values for the Lewis factor in air–water systems range from *Le*_f_ = 0.89 [30] to *Le*_f_ = 1.3 [36].

As a measure for the degree of humidification, the humidifier efficiency is defined as the ratio of the actual humidity ratio difference to the maximum possible humidity ratio difference (Eq. (22)):

(22) $$\eta_{\text{h}}=\frac{\omega_{\text{o}}-\omega_{\text{i}}}{\omega_{\text{sat}}-\omega_{\text{i}}}\cdot 100$$

## 3 Experimental details

### 3.1 Experimental setup

**Fig. 4:**
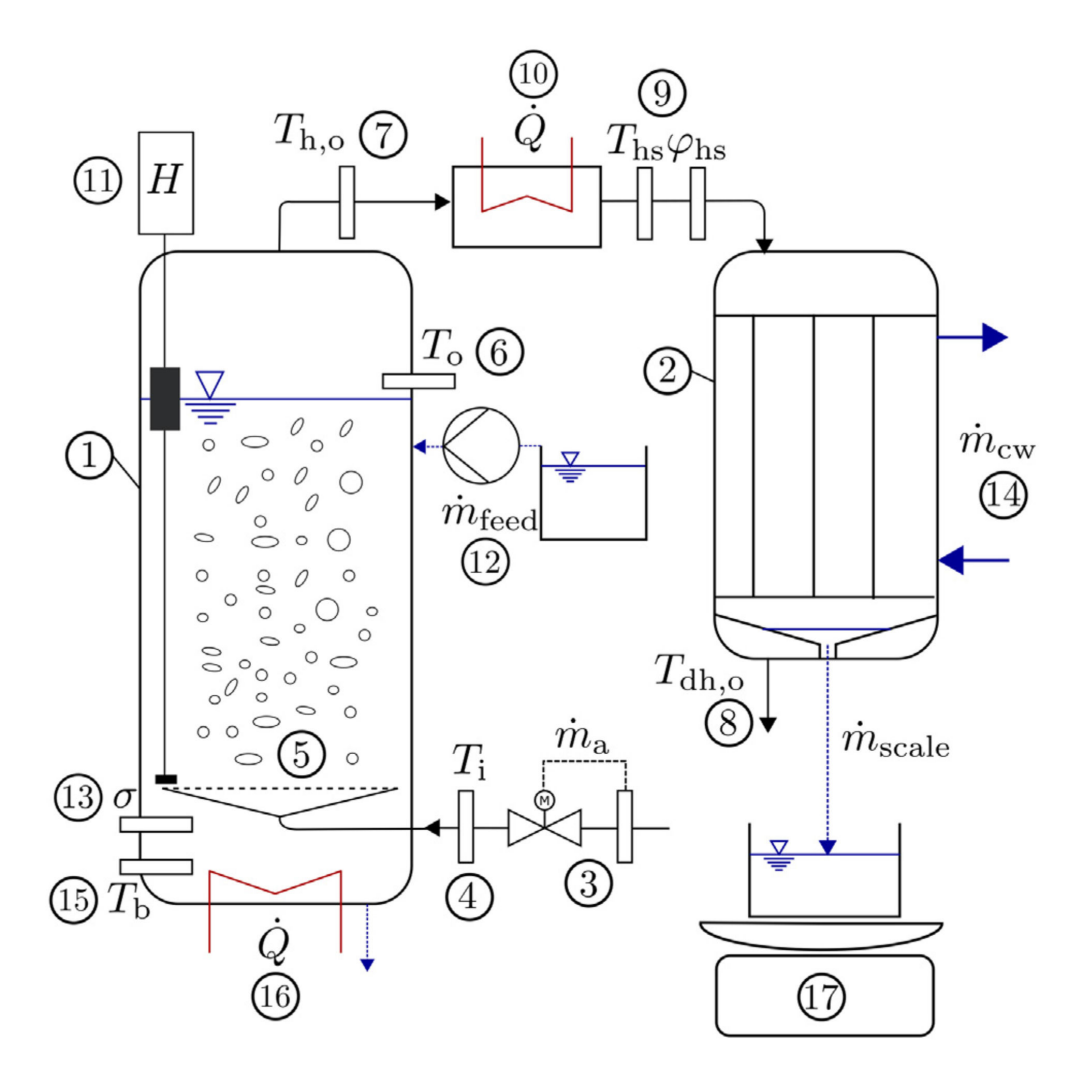
Experimental test setup for humidification measurements: (1) BCH, (2) fin and tube heat exchanger, (3) flow meter, (4,6,7,8,15) PT1000 resistance thermometers, (5) sparger assembly, (9) capacitive humidity sensor, (10) heating line, (11) liquid height sensor, (12) dosage pump, (13) electrical conductivity sensor, (14) cooling water cycle, (16) heating cartridges and (17) digital scale.

Our HDH test setup is visualized in Fig. 4 and consists of a BCH for humidification and a fin and tube heat exchanger for dehumidification.

The BCH (1) is built of acrylic glass cylinders and stainless steel parts with an inner diameter of *d* = 14 cm. The air mass flow ${\dot{m}}_{\text{a}}$ is set by a flow meter (3) before the air enters the humidifier through a sparger assembly (5). A sparger plate with an orifice diameter of *d*_0_ = 1 mm is used for all measurements. The air temperature is measured at the humidifier inlet *T*_i_ (4), directly above the surface of the liquid column *T*_o_ (6), at the humidifier outlet *T*_h,o_ (7) and at the dehumidifier outlet *T*_dh,o_ (8) with resistance thermometers. Furthermore, the air state after humidification is evaluated using a capacitive humidity sensor measuring temperature *T*_hs_ and relative humidity *φ*_hs_ (9). Due to reported difficulties with measuring humidity at the humidifier outlet [29,32], a heating line (10) is installed which lowers the relative humidity of the air stream while maintaining a constant humidity ratio. This allows for a more reliable humidity measurement and calculations of the theoretical relative humidity at positions (6) and (7) with the assumption of a constant humidity ratio. The liquid height *H* is measured by a floater-based sensor (11) and maintained by a dosage pump (12). An electrical conductivity sensor *σ* (13) is used to measure the salinity of the liquid phase. A separate water cycle ${\dot{m}}_{\text{cw}}$ (14) is used for cooling the air stream. The liquid temperature *T*_b_ is measured by a resistance thermometer (15) and controlled by heating cartridges (16). To ensure that condensation of the air stream due to heat loss is minimized, thermal insulation is applied to the BCH and the air pipes before the heating line. In the fin and tube heat exchanger, the air stream is cooled and the condensate is collected and continuously weighed by a digital scale, resulting in the condensate mass flow ${\dot{m}}_{\text{scale}}$ (17).

The liquid temperature *T*_b_, the superficial air velocity *υ*_as_ and the liquid height *H* are varied in the measurement ranges listed in Table 1.

### 3.2 Experimental procedure

By varying the operating parameters in the listed ranges of Table 1, the impact of the liquid temperature, the superficial air velocity, the liquid height, and the salinity on the volumetric transfer coefficients are assessed. For all measurements conducted, data is logged in 15 s intervals for 30 min in steady-state operation.

To enable a more detailed analysis and accurately determine the so-far unknown air state after humidification, we use different assumptions for this air state and compare the respective estimations of the system productivity with the actual productivity. The assumptions used for this purpose are listed in Table 2.

The estimated amount of condensate ${\dot{m}}_{\text{scale}}$ produced can be calculated by

(23) $${\dot{m}}_{\text{calc}}={\dot{m}}_{\text{a}}\cdot \left(\omega_{\text{dh},\text{i}}-\omega_{\text{dh},\text{o}}\right),$$

with *ω*_dh,i_ and *ω*_dh,o_ referring to the humidity ratios of air at the dehumidifier inlet and outlet. The humidity ratio at the dehumidifier outlet *ω*_dh,o_ can be calculated using the air outlet temperature *T*_dh,o_, as the air stream is always saturated at this position. The humidity ratio at the dehumidifier inlet *ω*_dh,i_ is calculated based on the assumptions listed in Table 2.

The humidity ratio of air is calculated by

(24) $$\omega =0.622\cdot \frac{\varphi \cdot p_{\text{v}}}{p_{\text{atm}}-\varphi \cdot p_{\text{v}}},$$

with *φ* referring to the relative humidity of air, *p*_v_ referring to the vapor pressure of water (calculated utilizing the Antoine equation), and *p*_atm_ referring to the atmospheric pressure of 101 325 Pa.

To quantify the impact of salinity on the humidification process, the vapor pressure of saline water is needed. We use a correlation of Nayar et al. [37] as it is suitable for the salinity and temperature ranges used in this study:

(25) $$ln\left(p_{\text{v},\text{sw}}/p_{\text{v}}\right)=-4.5818\cdot 10^{-4}\cdot S-2.0433\cdot 10^{-6}\cdot S^{2},$$

with *S* representing the salinity in g_NaCl_/kg_sw_ and *p*_v,sw_ representing the vapor pressure of saline water. For all measurements except for the measurement series to quantify salinity’s impact, tap water is used as the liquid phase.

### 3.3 Sensors and error analysis

Table 3 lists the sensors used with their associated measurement ranges and uncertainties.

For figures displaying relative changes of values, the standard deviation of the mean value is calculated using Eq. (26):

(26) $$s_{\bar{\text{x}}}=\frac{s_{\text{x}}}{\sqrt{n}}$$

with *s*_x_ representing the standard deviation, *x* representing the measured variable and *n* representing the number of measurements within the measurement period. As the standard deviations of the mean value are negligibly small for the measurements conducted in this study, they are not depicted in figures that display relative changes.

For figures that display absolute values, error bars indicate the uncertainty of the measurement instruments. For derived values, error propagation is used to calculate the standard deviation (see Eq. (27)):

(27) $$s_{\text{F}}=\sqrt{{\left(\sum_{\text{i}}\frac{\partial F}{\partial x_{\text{i}}}\right)}^{2}}s_{\text{i}}^{2}$$

with *x*_i_ denoting independent variables with their respective standard deviation *s*_i_ and function sensitivity *∂F*/*∂x*_i_.

## 4 Results and discussion

### 4.1 Air state at the humidifier outlet

**Fig. 5:**
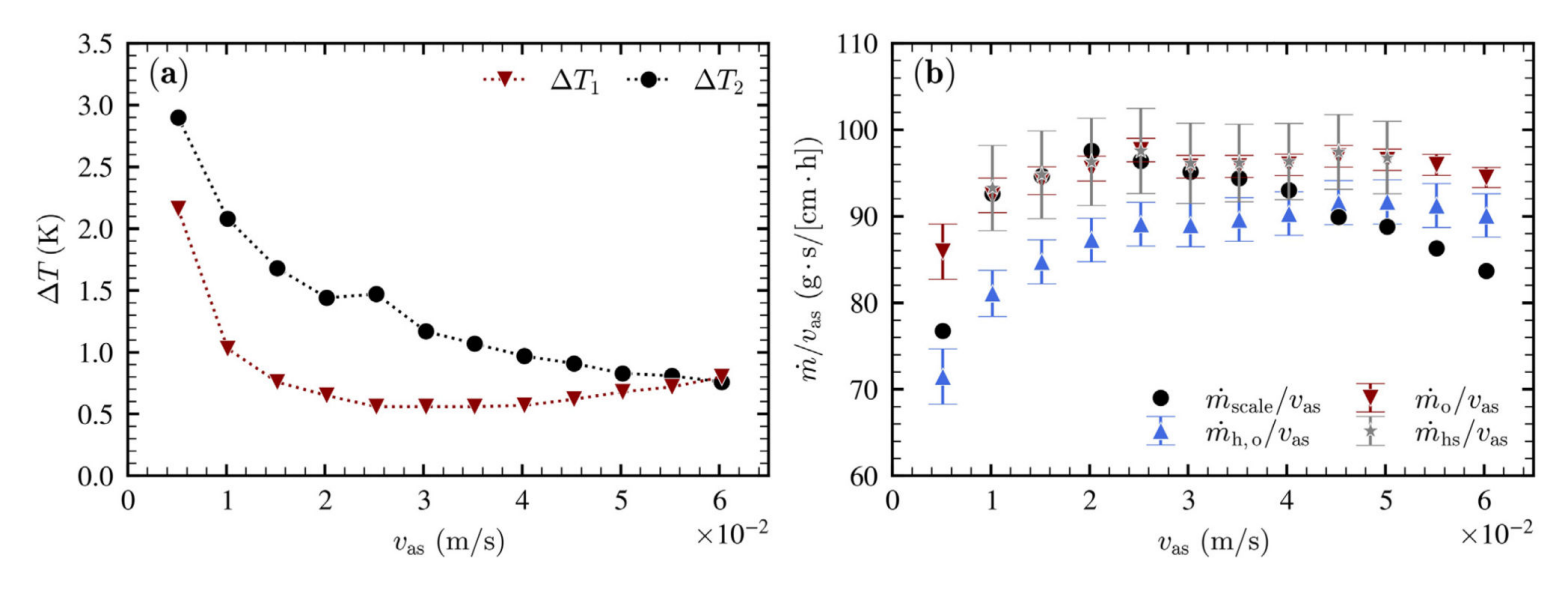
(a) Selected temperature differences with dependence on the superficial air velocity and (b) different estimations of productivity in comparison with the measured amount of produced condensate, measurements conducted at *T*_b_ = 60 °C and *H* = 80 mm.

To determine the air state after humidification, measurements at *T*_b_ = 60 °C, *H* = 80 mm with superficial air velocities between *υ*_as_ = 0.5 cm/s and 6.0 cm/s in steps of 0.5 cm/s are conducted and analyzed. In Fig. 5(a), two temperature differences are investigated:

- Δ*T*_1_: The difference between the liquid temperature *T*_b_ (15) and the air temperature at the liquid surface *T*_o_ (6)
- Δ*T*_2_: The difference between the air temperature at the liquid surface *T*_o_ (6) and the air temperature at the humidifier outlet *T*_h,o_ (7)

In Fig. 5(b), estimations of the productivity are depicted and compared to the measured amount of condensate produced. The underlying assumptions for the estimations are listed in Table 2. To simplify the comparisons, all productivities are divided by the set superficial air velocity in cm/s.

As can be seen in Fig. 5(a), the small superficial air velocities show that an increase in air velocity leads to a significant decrease in the temperature difference *ΔT*_1_. This is due to an improved heat transfer in the bubble column with respect to increasing turbulence. For air velocities higher than 4 cm/s, the temperature difference *ΔT*_1_ starts increasing since the residence time of the air stream in the liquid column is reduced with increasing air velocity. It is also evident that the temperature difference *ΔT*_2_ is reduced with superficial air velocity. A higher mass flow leads to an increase in the transported thermal mass and therefore to a lower temperature difference, even though heat loss is increased due to a higher Reynolds number.

The findings with respect to Fig. 5(b) can be summarized as follows:

- **The air stream is supersaturated at the humidifier outlet.** It can be seen that for superficial air velocities of up to *υ*_as_ = 4 cm/s, the measured productivity ${\dot{m}}_{\text{scale}}$ is higher than the calculated estimation ${\dot{m}}_{\text{h},\text{o}}$. According to Fig. 5(a), the air is cooled from the liquid surface to the humidifier outlet from 0.5 to 3 K. If the air stream is already close to saturation at the liquid surface, this cooling results in the condensation of water vapor. As this water vapor is not separated from the air stream, but instead carried with the air stream, the air is in a supersaturated state.
- **The air stream is saturated at the liquid surface** since the estimations for ${\dot{m}}_{\text{o}}$ and ${\dot{m}}_{\text{hs}}$ are almost identical for all measurements.
- The decrease of the measured productivity ${\dot{m}}_{\text{scale}}$ at high superficial air velocities can only be explained by a **decreasing dehumidifier effectiveness for an increasing superficial air velocity**, as superficial air velocities higher than *υ*_as_ = 4 cm/s showed that the measured productivity starts decreasing significantly. The system temperatures and the humidity sensor do not indicate this substantial decrease.

**Fig. 6:**
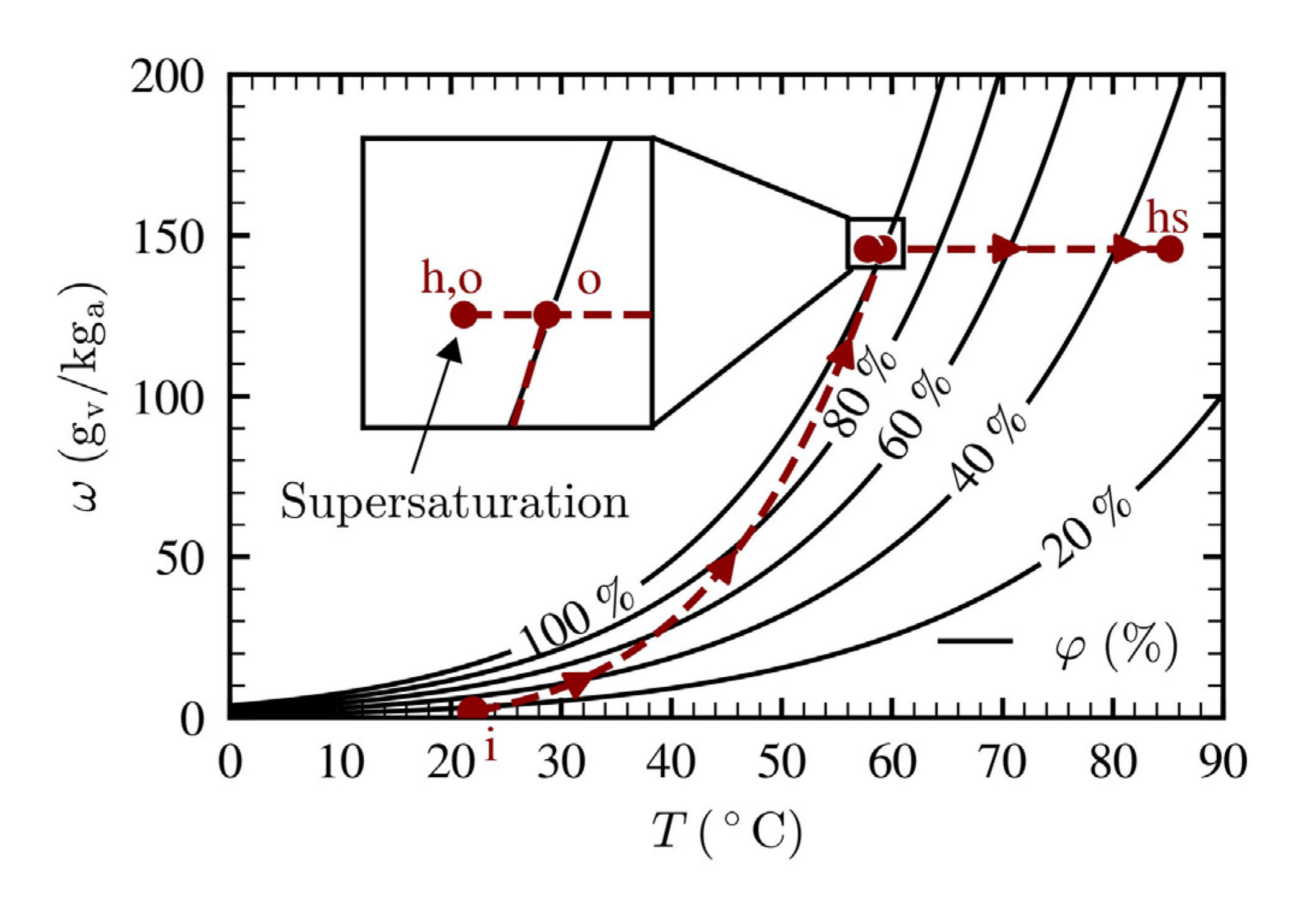
Occurring air states in a humidification process, measurement conducted at *T*_b_ = 60 °C, *H* = 80 mm and *υ*_as_ = 2 cm/s.

For a better understanding of the previous results, the respective air states of the process are visualized in Fig. 6 for a single parametric setting.

The different process steps according to Fig. 6 can be summarized as follows:

- i → o: Humidification of the air stream from the humidifier inlet to the liquid surface, which the air exits in a saturated state. The visualized process path is symbolic and is dependent on the extent of the air–liquid heat and mass transfer.
- o → h, o: Cooling of the air stream as a result of radial heat loss. Condensation of water vapor occurs, liquid droplets are formed and carried with the air stream. The moist air is therefore in a supersaturated state.
- h, o → hs: Heating of the air stream using the heat line after the humidifier. The relative humidity of the air stream is significantly reduced while maintaining the humidity ratio.

### 4.2 Comparison of methods to determine the transfer coefficients

In previous studies volumetric transfer coefficients have been calculated for single measurements [31,33]. This results in an apparent dependence of the volumetric transfer coefficients on the liquid height and turns out to be inaccurate if measurements are conducted in parametric ranges, where changes of humidity ratio are smaller than the measurement uncertainty. This is the case for liquid heights higher than *H* = 0.05 m. It also explains why Zizka et al. [31] found that the impact of liquid height on the volumetric heat transfer coefficient can be compensated by using aeration as a measure of air flow.

**Fig. 7:**
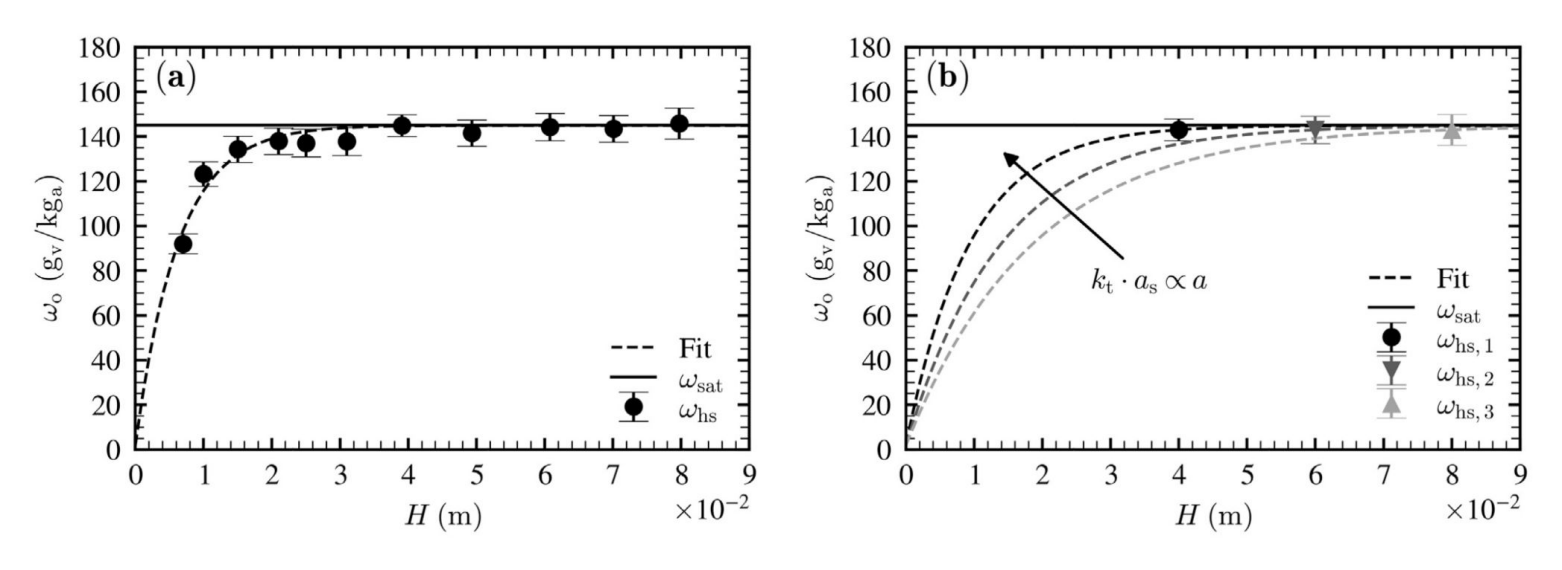
(a) Determination of the volumetric mass transfer coefficient by nonlinear regression using multiple measurement points and (b) determination of the volumetric mass transfer coefficient using single measurements, which leads to a false dependence of the volumetric mass transfer coefficient on the liquid height, measurements conducted at *T*_b_ = 60 °C and *υ*_as_ = 2 cm/s.

We state that the most accurate method to determine the volumetric heat and mass transfer coefficients is to apply nonlinear regression to multiple measured values of the outlet humidity ratio under various operating conditions. In Fig. 7, the two methods are compared for a series of measurements. Fig. 7(a) shows the nonlinear regressive fit through multiple measurement points, whereas Fig. 7(b) shows the calculation of the volumetric transfer coefficients based on single measurements. This is equivalent to a nonlinear fit through each measurement point and results in an apparent dependence of volumetric mass transfer coefficient *k*_t_*a*_s_ on the liquid height (i.e. if the liquid height is doubled and the outlet humidity ratio stays the same, as it is the case for *ω*_hs,1_ and *ω*_hs,3_, the volumetric mass transfer coefficient would decrease by a factor of 2).

On the other hand, using nonlinear regression on multiple measured values of different liquid heights results in a single value for parameter *a* and therefore for the volumetric mass transfer coefficient *k*_t_*a*_s_. Conclusively, it is necessary to measure in a parametric range where significant changes in the outlet humidity ratio take place and to take several measurements at different liquid heights to determine a single value for the volumetric heat or mass transfer coefficient.

### 4.3 Impact of operating parameters on heat and mass transfer

#### Salinity

**Fig. 8:**
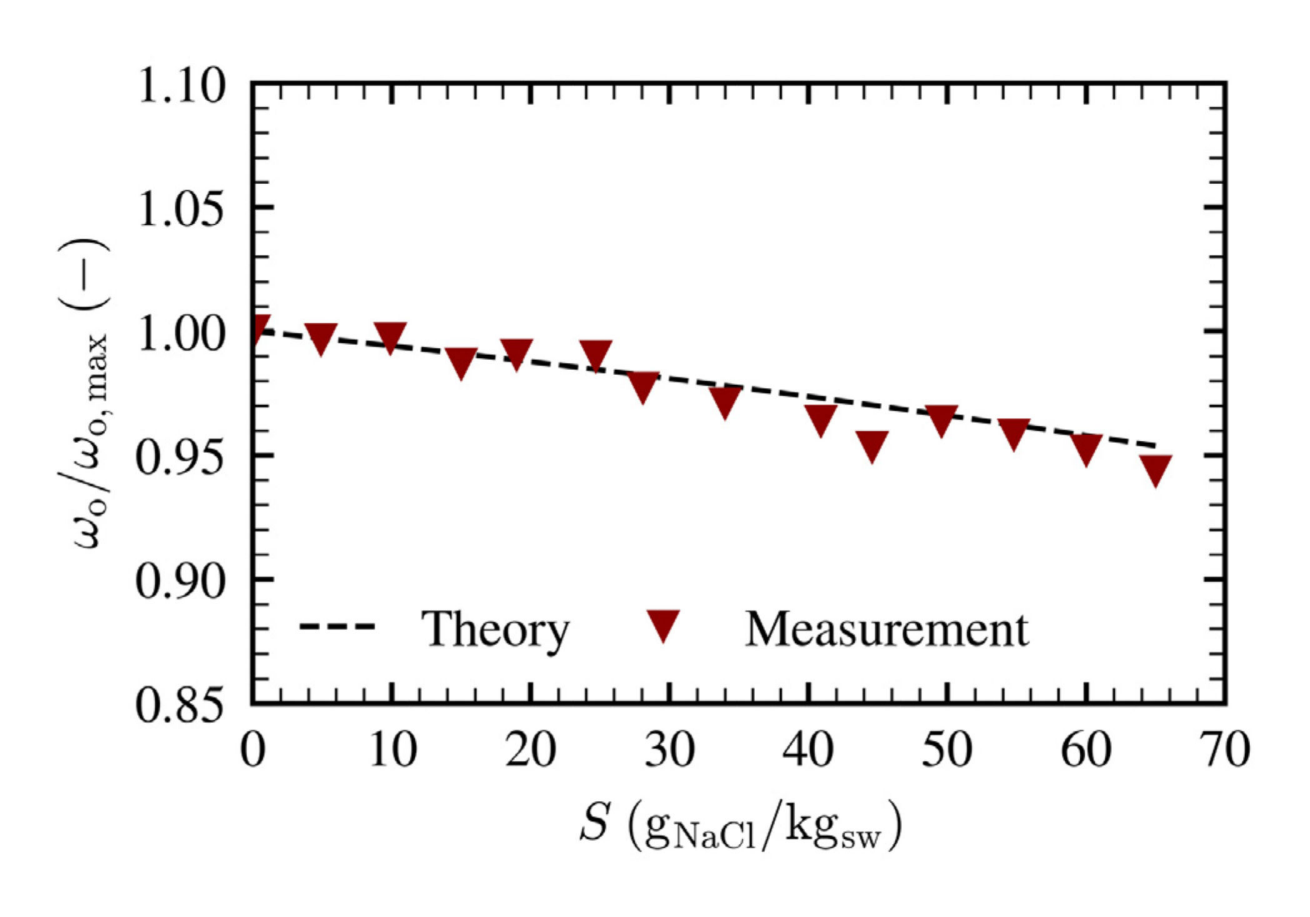
Measured and calculated relative change in humidity ratio for various salinities, measurements conducted at *T*_b_ = 60 °C, *υ*_as_ = 2 cm/s and *H* = 60 mm.

The impact of salinity on the heat and mass transfer and the corresponding outlet air state can be explained using Fig. 8. The measured relative change in humidity ratio (measured with the humidity sensor (9)) is compared to the theoretical relative decline in humidity ratio for an increasing salinity in accordance with Eq. (25).

There is a similar trend between the measured and the theoretical change in humidity ratio. Consequently, from the investigated measurement range it can be stated that the heat and mass transfer are unaffected by the variation in salinity and that the vapor pressure reduction is responsible for occurring changes in productivity. Small fluctuations of the measured humidity ratio *ω*_hs_ are attributed to minor changes of the liquid temperature *T*_b_ and of the corresponding air surface temperature *T*_o_.

#### Liquid temperature

**Fig. 9:**
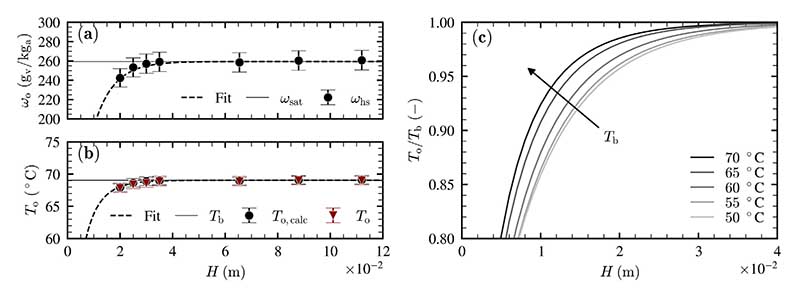
(a) Outlet humidity ratio *ω*_o_ with dependence on liquid height for *T*_b_ = 70 °C, (b) Outlet air temperature *T*_o_ with dependence on liquid height for *T*_b_ = 70 °C and (c) nonlinear regressive fit for various liquid temperatures, measurements conducted at *υ*_as_ = 2 cm/s.

To characterize the impact of liquid temperature on the volumetric transfer coefficients, nonlinear regression is applied to measurements with the liquid height varying between *H* = 20 mm and *H* = 105 mm. The liquid temperature is varied between *T*_b_ = 50 °C and 70 °C in steps of 5 K. The measured humidity ratio *ω*_hs_ and the calculated and measured surface temperatures *T*_o,calc_ and *T*_o_ are depicted in Fig. 9(a) and (b) with dependence on the liquid height for a liquid temperature of *T*_b_ = 70 °C. The expected surface temperature *T*_o,calc_ is calculated based on the measured humidity ratio *ω*_hs_ and the assumption of saturated air. The nondimensional change of the air temperature at the liquid surface (*T*_o_/*T*_b_) is fitted by nonlinear regression and depicted for each investigated liquid temperature in Fig. 9(c).

An increase of the outlet humidity ratio and the outlet temperature with liquid height is evident (see Fig. 9(a) and (b)). In addition to that, the expected surface temperature *T*_o,calc_ agrees with the measured surface temperature *T*_o_ with excellent accuracy.

According to Fig. 9(c), the temperature and the corresponding humidity ratio of the air are increasing faster for higher liquid temperatures. As a result, the volumetric transfer coefficients also increase as the liquid temperature increases.

Nonlinear regression yields the exponential fit through the measurement points and allows us to calculate the volumetric heat and mass transfer coefficients. These are listed in Table 4 for all investigated temperatures.

According to Table 4, there is an increase of both volumetric transfer coefficients with increasing liquid temperature. Lewis factors vary slightly with the liquid temperature but also remain higher than unity for the investigated temperatures. While Narayan et al. [30] suggest the Lewis factor to be of approximately 0.89−0.92 for air–water systems, as it is recommended for wet cooling towers [36], Srithar and Rajaseenivasan suggest it to be equal to 1 [28]. For the measurements conducted in this study, the Lewis factor always lies between 1 and 1.2.

#### Superficial air velocity

**Fig. 10:**
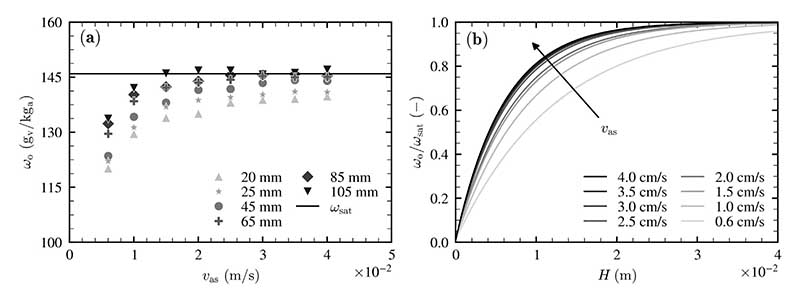
(a) Outlet humidity ratio *ω*_o_ with dependence on superficial air velocity for various liquid heights and (b) Nondimensional change of air humidity ratio at the liquid surface with dependence on liquid height for various superficial air velocities, measurements conducted at *T*_b_ = 60 °C.

The impact of superficial air velocity on the volumetric transfer coefficients is characterized similarly by variations of the liquid height at several values of the superficial air velocity. The outlet humidity ratio *ω*_o_, being dependent on the superficial air velocity, is depicted for several liquid heights in Fig. 10(a). The nondimensional change of the outlet humidity ratio (*ω*_o_/*ω*_sat_) is fitted by nonlinear regression and displayed for all investigated superficial air velocities in Fig. 10(b). The uncertainties of the measurements are not depicted in this figure to enable a better comparison (see Fig. 10(a)).

According to Fig. 10(a), the most significant increase in the outlet humidity ratio is evident for superficial air velocities up until *υ*_as_ = 2 cm/s. An increase in the turbulence is responsible for this increase. For superficial air velocities higher than *υ*_as_ = 2 cm/s, the outlet humidity ratio is increased slightly and even remains steady for higher liquid heights. As can be seen in Fig. 10(b), *ω*_sat_ is reached more rapidly for an increase in the superficial air velocity.

Nonlinear regression is used to evaluate the volumetric heat and mass transfer coefficients with dependence on superficial air velocity. These are listed in Table 5.

According to Table 5, both of the volumetric heat and mass transfer coefficients are significantly increased with an increase in the superficial air velocity. On the other hand, the Lewis factor decreases with an increase in superficial air velocity. The values of the Lewis factor are again higher than unity.

### 4.4 Parametric correlations for the volumetric transfer coefficients

The calculated results for the volumetric heat and mass transfer coefficient are fitted in accordance with Eq. (28) using multivariate nonlinear regression.

(28) $$\begin{array}{l} h_{\text{t}}a_{\text{s}}=C_{1}\cdot T_{\text{b}}{\;}^{C_{2}}\cdot \upsilon_{\text{as}}{\;}^{C_{3}}\\ k_{\text{t}}a_{\text{s}}=C_{1}\cdot T_{\text{b}}{\;}^{C_{2}}\cdot \upsilon_{\text{as}}{\;}^{C_{3}} \end{array}$$

Correlations for the volumetric heat and mass transfer coefficient and the coefficient of determination *R*^2^ are given in Table 6.

Both of the volumetric transfer coefficients are increasing with the liquid temperature and the superficial air velocity. The volumetric heat transfer coefficient is increased almost quadratically with the liquid temperature and more than proportional with the superficial air velocity. On the other hand, the volumetric mass transfer coefficient is increased approximately linearly with both operating parameters.

The presented correlations are valid for a liquid temperature between 50 and 70°C, a superficial air velocity between 0 and 0.04 m/s and a liquid height between 0 and 0.1 m. As for the superficial air velocity, a decrease in the outlet humidity ratio is expected for superficial air velocities higher than 0.04 m/s, and correspondingly, the exponent *C*_3_ will reduce to less than 1 for air velocities in this range.

To calculate air humidity ratio at the outlet of a BCH, the volumetric mass transfer coefficient has to be calculated using Eq. (28). Subsequently, Eqs. (12) and (13) can be used to calculate the outlet air state for a specific liquid height.

### 4.5 Humidifier efficiency chart

For all measurements conducted at *T*_b_ = 60 °C, the humidifier efficiency is calculated by Eq. (22) and fitted using nonlinear regression (*R*^2^ = 0.989) to create a humidifier efficiency chart with respect to the superficial air velocity and the liquid height. For the creation of this efficiency chart, two additional measurement series are considered at liquid heights below *H* = 0.02 m. However, they are not used to calculate the volumetric transfer coefficients, as for low liquid heights, slight differences in the liquid height lead to significant changes in the humidity ratio and therefore induce more considerable uncertainties. This should also be considered when interpreting the efficiency chart for low values of the liquid height and the superficial air velocity. Although this efficiency chart is valid only for a liquid temperature of *T*_b_ = 60 °C, it is quite insensitive to changes in the liquid temperature.

**Fig. 11:**
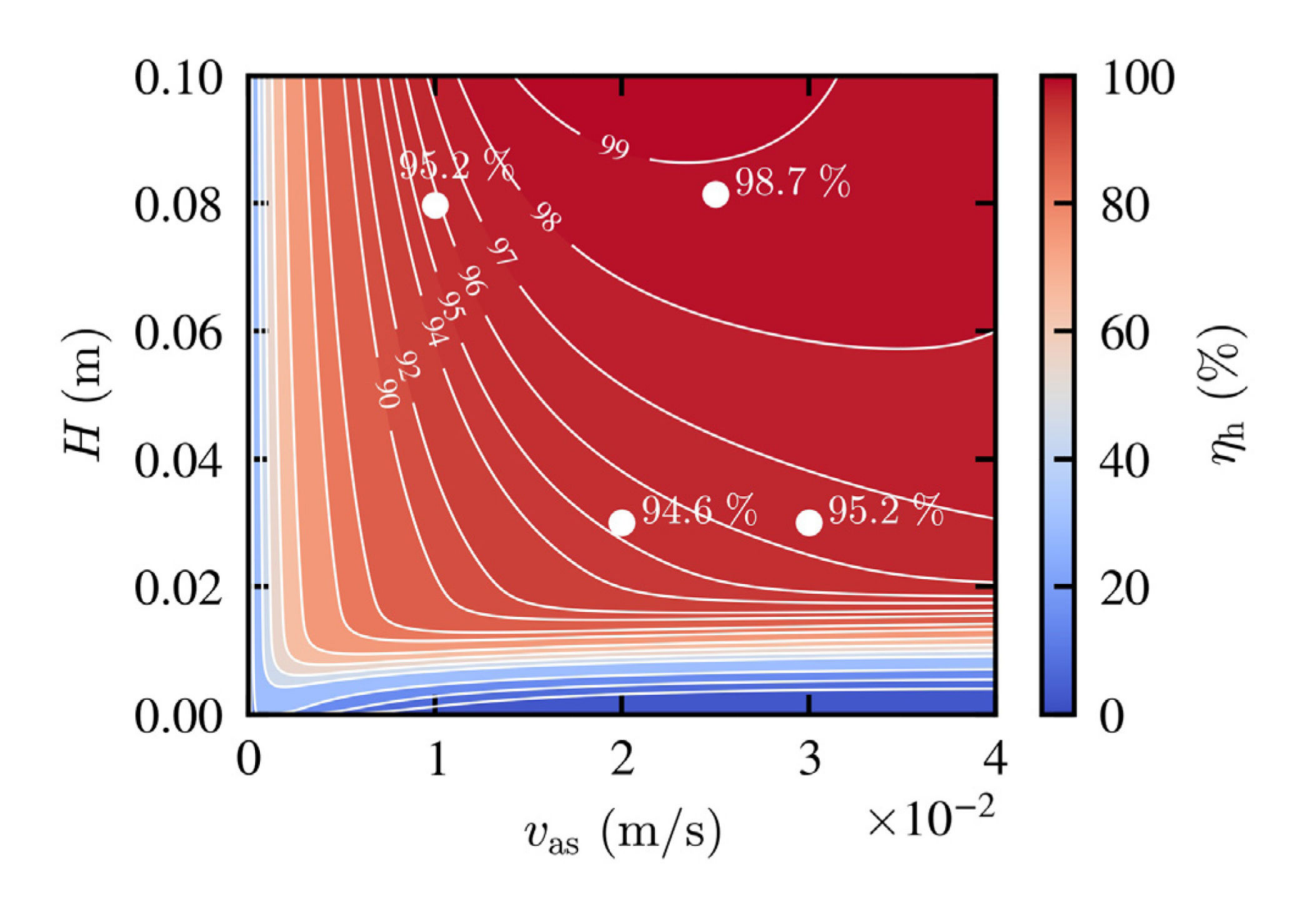
Humidifier efficiency plot with respect to the superficial air velocity and the liquid height, four additional measurements are displayed as test data for validation purposes, measurements conducted at *T*_b_ = 60 °C.

Fig. 11 allows for the appropriate selection of operating conditions to reach a required humidifier efficiency. To validate the accuracy of the efficiency plot, four additional measurements are carried out and visualized in Fig. 11. These measurements are not used for the fit itself and can therefore be considered as test data.

According to Fig. 11, the absolute error of the humidifier efficiency is below 1% for high liquid heights and below 2% for low liquid heights. Superficial air velocities below *υ*_as_ = 1 cm/s and liquid heights below *H* = 20 mm are expected to produce higher absolute errors, however. Therefore, the main use of this efficiency chart lies in the recommendation of parametric ranges for industrial applications and future scientific studies.

For the industrial application, the liquid height of a BCH should be reduced as far as possible. As can be seen in Fig. 11, with moderate superficial air velocities, a liquid height of *H* = 50 mm is sufficient, to reach a humidifier efficiency of at least *η*_h_ = 95%. Future scientific studies of BCHs, on the other hand, should be conducted in parametric ranges, where significant changes of outlet humidity ratio are to be expected (i.e. *H* < 0.04 m and *υ*_as_ < 3 cm/s).

## 5 Conclusion

Using a systematic approach, the humidification of air in a bubble column is mathematically modeled and experimentally investigated. Our findings can be summarized as follows:

- The air is proven to be in a saturated state (*φ* = 100%) at the liquid surface and supersaturated at the humidifier outlet for various operational settings.
- For the first time, the impact of the liquid height is accurately characterized by measurements at liquid heights below 0.1 m. In contrast to previous studies, the volumetric transfer coefficients are determined by nonlinear regression using multiple measured values at different liquid heights instead of single measurements.
- The volumetric transfer coefficients are shown to increase with both the liquid temperature and the superficial air velocity.
- Increasing the salinity decreases the saturation vapor pressure of moist air. This theoretical decrease corresponds to the measured decrease in the humidity ratio at the liquid surface of the humidifier.
- Correlations for the volumetric heat and mass transfer coefficients with dependence on the main operating parameters are presented, as well as an efficiency chart to determine the expected humidifier efficiency with respect to these parameters.

Our results significantly increase the knowledge of different phenomena accompanying humidification in bubble columns. The proof that the air stream is saturated and close to the liquid temperature simplifies the design of bubble columns for various applications. For future scientific studies, we recommend investigating liquid heights below *H* = 50 mm, as significant changes of the air stream humidity ratio occur in this range. To eventually derive correlations for the heat and mass transfer coefficient in non-volumetric form, analyses of the bubble size and the gas holdup should also be conducted for these low liquid heights, resulting in the interfacial area between the air stream and the liquid phase. As low liquid heights are very challenging to measure with a sufficient accuracy, experimental setups should be specifically designed for this task.

As for the application, we recommend BCHs to be operated with a liquid height of *H* = 50 mm and with superficial air velocities between *υ*_as_ = 2 and 6 cm/s, as the humidifier efficiency will already exceed *η*_h_ = 95% and the air-side pressure loss due to the column height can be minimized.

## Figures and Tables

**Table 1:** Operating parameters with their respective measurement range and step size.

Parameter	Variation range	Step size
*T*_b_	50–70 °C	5 K
*υ*_as_	1.0–6.0 cm/s	0.5 cm/s
*H*	20–100 mm	5 and 20 mm
*S*	0–70 g_NaCl_/kg_sw_	5 g_NaCl_/kg_sw_

**Table 2:** Assumptions for estimations to determine the air state at the humidifier outlet.

Estimation	*φ*	*T*
	%	°C
${\dot{m}}_{\text{o}}$	100	*T*_o_
${\dot{m}}_{\text{h,o}}$	100	*T*_h,o_
${\dot{m}}_{\text{hs}}$	*φ*_hs_	*T*_hs_

**Table 3:** Measurement sensors, measurement ranges and uncertainties.

Instrument	Measurement range	Uncertainty
Resistance thermometer class B (4,6,7,8,15)	0–100 °C	±[0.3 + 0.005 ⋅ T] °C
Resistance thermometer class AA (9)	−40–180 ° C	±[0.1 + 0.0017 ⋅ T] °C
Capacitive humidity sensor (9)	0–100 %RH	±[1 + 0.007 ⋅ *φ*] %RH
Float level liquid height sensor (11)	0 – 500 mm	±0.5 mm
Mass flow sensor (3)	0–10 ${\text{m}}_{\text{stp}}^{3}/\text{h}$	±0.01 ${\text{m}}_{\text{stp}}^{3}/\text{h}$
Digital scale (18)	0–3100 g	±0.1 g
Capacitive conductivity sensor (13)	0–500 mS/cm	±[2.5 + 0.005 ⋅ *σ*] mS/cm

**Table 4:** Volumetric transfer coefficients and Lewis factor for various liquid temperatures.

*T*_b_	*h*_t_ *a*_s_	*k*_t_ *a*_s_	*Le*_f_
°C	W/[m^3^ K]	kg/[m^3^ s]	−
50	3443	2.750	1.070
55	3736	2.853	1.070
60	4245	2.915	1.126
65	5206	3.563	1.052
70	5996	3.808	1.033

**Table 5:** Volumetric transfer coefficients and Lewis factors for various superficial air velocities.

υ_as_	*h*_t_ *a*_s_	*k*_t_ *a*_s_	*Le*_f_
cm/s	W/[m^3^ K]	kg/[m^3^ s]	−
0.6	891	0.587	1.172
1.0	2008	1.334	1.162
1.5	3296	2.391	1.065
2.0	4503	3.361	1.035
2.5	6298	4.683	1.039
3.0	7897	5.838	1.044
3.5	9074	6.999	1.001
4.0	10 493	8.160	0.993

**Table 6:** Correlation coefficients and coefficient of determination for volumetric heat and mass transfer coefficient.

Variable	*h*_t_ *a*_s_	*k*_t_ *a*_s_
	W/[m^3^ K]	kg/[m^3^ s]
*C*_1_	459.6	9.136
*C*_2_	1.732	1.025
*C*_3_	1.225	1.334
*R* ^2^	0.995	0.994

## Nomenclature

**Acronyms**
BCH: Bubble column humidifier
HDH: Humidification–dehumidification

**Greek Symbols**
*η*_h_: Humidifier efficiency (%)
ω: Humidity ratio (−)
σ: Electrical conductivity (S/m)
φ: Relative humidity (%)

**Nondimensional Numbers**
*Le*_f_: Lewis factor (−)

**Physical Properties**
*Δh*_v_: Enthalpy of vaporization (J/kg)
$\dot{m}$: Mass flow (kg/s)
$\dot{Q}$: Heat flow (W)
*A*_c_: Cross-sectional area (m^2^)
*a*_s_: Specific interfacial area (1/m)
*C**: Moist air heat capacity rate (W/K)
$c_{\text{p}}^{*}$: Moist air specific heat capacity (J/[kg K])
*c*_p_: Specific heat capacity (J/[kg K])
*d*_0_: Orifice diameter (m)
*F*: Function (−)
*H*: Liquid height (m)
*h*: Specific enthalpy (J/kg)
*h**: Moist air specific enthalpy (J/kg)
*h*_t_: Heat transfer coefficient (W/[m^2^ K])
*k*_t_: Mass transfer coefficient (kg/[m^2^ s])
*n*: Number of measurements (−)
*P*: Equivalent perimeter (m)
*p*: Pressure (Pa)
*p*_v_: Vapor pressure (Pa)
*S*: Salinity (g_NaCl_/kg_sw_)
*s*: Standard deviation (−)
*T*: Temperature (°C)
*V*: Volume (m^3^)
*υ*: Velocity (m/s)

**Subscripts**
a: Air
as: Air superficial
atm: Atmospheric
b: Bulk liquid
c: Condensate
calc: Calculated estimation
cw: Cooling water
dh,i: Dehumidifier inlet
dh,o: Dehumidifier outlet
evap: Evaporated
feed: Feed liquid
h,o: Humidifier outlet
hs: Humidity sensor
ht: Heat transfer
i: Humidifier inlet
lat: Latent
max: Maximum
mt: Mass transfer
o: Liquid surface
sat: Saturated
sens: Sensible
stp: Standard temperature and pressure
sw: Saline water
x: At certain position
